# Effects of a recreational exercise program on digital addiction and sleep quality in children

**DOI:** 10.3389/fpsyg.2026.1869072

**Published:** 2026-07-01

**Authors:** Mehmet Altin

**Affiliations:** Department of Recreation, Faculty of Sport Sciences, Selçuk University, Konya, Türkiye

**Keywords:** 10–14 age group, digital addiction, recreational exercise, sedentary lifestyle, sleep quality

## Abstract

This study aimed to examine the effects of a structured recreational exercise program on digital addiction levels and sleep quality in children aged 10–14. A randomized controlled experimental design with repeated measures was employed. The sample consisted of 40 middle school students (20 girls and 20 boys), who were randomly assigned to a Recreational Exercise Group (REG, *n* = 20) or a Control Group (CG, *n* = 20). The intervention involved an 8-week recreational exercise program, including a 2-week adaptation phase followed by a 6-week main implementation period, conducted three times per week. Digital addiction and sleep quality were assessed at three time points—pre-test, post-test, and 6-week follow-up—using the Digital Addiction Scale for Adolescents and the Sleep Quality Scale and Sleep Variables Questionnaire. Data were analyzed using a 2 × 3 mixed-design analysis of variance (ANOVA). The results revealed significant group × time interaction effects for both digital addiction and sleep quality (*p* < 0.01). Digital addiction levels in the REG decreased significantly from pre-test to post-test and remained stable at follow-up, whereas no significant changes were observed in the control group. Similarly, sleep quality improved significantly in the REG following the intervention, with the observed improvements maintained at follow-up. Effect size estimates indicated large effects for both outcomes. These findings suggest that regular participation in structured recreational exercise programs is an effective and sustainable approach for reducing digital addiction and improving sleep quality in children aged 10–14, highlighting the potential of school-based recreational interventions in early adolescence.

## Introduction

In contemporary society, the rapid and pervasive spread of digital technologies has profoundly transformed individuals’ daily lives, social relationships, and leisure-time activities. As traditional forms of addiction (such as substance use and gambling) gradually give way to behavior-based addictions, problematic and excessive use of digital tools has emerged as a major public health concern. In this context, children and adolescents represent the most vulnerable groups, both because of their developmental characteristics and their intensive engagement with technology. Early adolescence, typically spanning the ages of 10–14, is marked by rapid biological, cognitive, and social changes that can heighten sensitivity to environmental influences and increase susceptibility to various risk behaviors, including digital addiction (Lissak, 2018). During this period, children strive to construct a coherent sense of identity and autonomy, while simultaneously seeking approval and belonging within their peer groups. The quality of parental and sibling relationships plays a decisive role in shaping identity development and social–emotional adjustment, and supportive family structures can serve as an important protective factor against maladaptive behavioral patterns (Crocetti et al., 2017).

When this developmental sensitivity coincides with the ease of access, constant connectivity, and stimulating nature of digital environments, the likelihood of excessive and uncontrolled digital engagement increases. Compared with other forms of addiction, digital addiction is characterized by its high accessibility, low immediate cost, and relatively weak external control mechanisms, which together make it more insidious and more difficult to regulate. Children can frequently access digital devices (such as smartphones, tablets, and computers) without strict supervision, enabling prolonged screen time and reinforcing compulsive use patterns. Digital addiction has been associated not only with physical health problems but also with a wide range of negative emotional, cognitive, and behavioral outcomes (Nakshine et al., 2022). Excessive screen time contributes to sedentary lifestyles, which are linked to obesity, musculoskeletal problems, and cardiovascular risk factors. Beyond physical consequences, problematic digital use is associated with increased levels of anxiety, depressive symptoms, irritability, attention problems, and academic difficulties (Eryılmaz and Çukurluöz, 2018). Recent studies have shown that children and adolescents are increasingly drawn to digital games, social media platforms, and online content (Akçayır, 2013), and that digital addiction has become a growing risk for both children and adults, with prevalence rates steadily rising (Altınok, 2021). In parallel, the ease of access to the internet and the lack of effective monitoring mechanisms can expose children to various forms of online victimization and abuse, thereby increasing their vulnerability (Arslan, 2020).

The growing prevalence of digital addiction has also led to substantial changes in children’s daily routines and lifestyle habits, particularly by encouraging withdrawal from face-to-face social interactions and a preference for technology-based activities. Children with high levels of digital addiction are more likely to prioritize virtual interactions, online games, and social media use over in-person relationships and outdoor play (Nakshine et al., 2022; Lissak, 2018). However, early adolescence is a critical period for developing social skills, emotional regulation abilities, and interpersonal competence. During this stage, participation in peer groups, engagement in cooperative activities, and involvement in structured social contexts contribute to healthy psychosocial development. When technology-based groups and online communities dominate children’s social lives, opportunities for practicing essential social and emotional skills may be substantially reduced. Yılmaz (2019) argued that digital technologies can transform individuals’ perceptions of reality, leading children to experience online environments more intensely and to attribute greater importance to virtual achievements and relationships than to real-life experiences. From a developmental perspective, this shift can delay or hinder the fulfillment of basic physiological and psychosocial needs, resulting in irregular eating habits, impaired sleep patterns, reduced physical activity, and diminished psychological well-being. Over time, these disruptions may predispose children to various health problems and maladaptive behaviors later in life.

Among the domains most critically affected by digital addiction is sleep regulation. Sleep is a fundamental biological necessity that supports children’s physical growth, brain maturation, cognitive functioning, and emotional stability. Adequate and good-quality sleep is essential for attention, memory consolidation, learning processes, and behavioral regulation. Disturbances in sleep patterns can have wide-ranging negative effects on children’s academic performance, mental health, social–emotional development, and overall quality of life. Schlieber and Han (2021) emphasized that children’s sleep habits are shaped by multiple interacting factors, including family routines, parental monitoring, bedroom environment, and cultural norms. Hirshkowitz et al. (2015) reported that sleep duration tends to decrease with age and that developmental changes can alter sleep architecture and circadian rhythms. According to the National Sleep Foundation, the recommended sleep duration for children aged 6–13 years is 9–11 h per night, while George and Davis (2013) suggested that approximately 9–10 h of sleep per day is optimal for adolescents. Notably, recent studies have demonstrated a significant association between pre-bedtime use of mobile phones and reduced sleep duration, with late-night screen use and exposure to blue light linked to delayed sleep onset and shorter total sleep time (Cain and Gradisar, 2010; Hale and Guan, 2015). Arora et al. (2014) reported that children and adolescents who engage in late bedtime behaviors, such as prolonged mobile phone or tablet use, may experience an average sleep loss of around 45 min per night. Beyond the detrimental effects of screen exposure on sleep, the physiological pathways through which physical exercise enhances sleep quality merit explicit consideration. Regular aerobic exercise has been shown to promote deeper and more restorative sleep through several interacting mechanisms. First, exercise accelerates the accumulation of adenosine—a sleep-promoting neuromodulator—in the brain, thereby increasing homeostatic sleep pressure and facilitating earlier sleep onset (Youngstedt, 2005). Second, the transient rise in core body temperature during exercise is followed by a compensatory decline in the post-exercise period, a thermoregulatory process associated with the initiation and consolidation of slow-wave sleep (Horne and Staff, 1983). Third, participation in structured physical activity has been linked to reductions in pre-sleep cognitive arousal and rumination, which are common barriers to sleep onset in children and adolescents experiencing psychological stress (Kredlow et al., 2015). Finally, regular exercise contributes to the stabilization of circadian rhythms by reinforcing the amplitude of the sleep–wake cycle, thereby reducing sleep onset latency and nocturnal awakenings (Reid et al., 2010). Taken together, these mechanisms provide a robust biological rationale for expecting that a structured exercise intervention will produce measurable improvements in sleep quality among children with high digital device exposure.

Considering digital addiction together with sleep disturbances allows for a more comprehensive understanding of the multifaceted risks faced by children and underscores the need for integrated, solution-oriented interventions. It is increasingly recognized that multidimensional approaches, rather than solely focusing on limiting screen time, are needed to support children’s healthy development. One promising approach involves physical activity–based games and recreational activities designed to offer enjoyable, socially engaging, and developmentally appropriate alternatives to screen-based leisure. Play is a natural and indispensable component of life for children aged 10–14 and occupies a central place in their cognitive, physical, and socio-emotional development. Structured and unstructured play activities allow children to explore their environment, test their limits, develop problem-solving skills, and learn social rules through direct experience. Taşkın and Özdemir (2018) reported that when children are encouraged to engage in physical activity compatible with their developmental characteristics, they participate more willingly, experience greater enjoyment, and display higher levels of success. In this context, play can be viewed not only as a source of entertainment but also as an educational tool that enables children to interpret, internalize, and adapt to their surroundings (Fanuscu, 1994). Within this context, it is important to distinguish between recreationally oriented activities and formally competitive sports as intervention modalities. While both forms of physical activity share aerobic and motor benefits, recreational and game-based programs offer several distinct advantages for children at risk of digital addiction. Competitive sports environments may introduce performance pressure, fear of failure, and social comparison, which can elevate cortisol levels and paradoxically heighten stress and anxiety in vulnerable adolescents (Scanlan and Passer, 1978). In contrast, recreational activities, particularly those grounded in the Teaching Games for Understanding (TGfU) framework, prioritize tactical enjoyment, intrinsic motivation, and inclusive participation over outcome-driven performance (Bunker and Thorpe, 1982). This approach lowers the barrier to entry for less physically confident children, sustains long-term engagement, and creates a psychologically safe environment in which social bonds can be organically formed. From a behavioral substitution perspective, the enjoyment and social fulfillment derived from recreational play may more effectively satisfy the emotional and social needs that children commonly seek to meet through digital environments, making recreational exercise a theoretically superior, and more ecologically valid, intervention for reducing digital dependency. In addition to fostering motor and cognitive development, play has been shown to exert positive effects on mental health by reducing stress, promoting positive emotions, and supporting resilience (Gray, 2011). Henricks (2014) emphasized that play provides individuals with unique experiential pathways and helps establish a behavioral trajectory that guides future actions and preferences. Organized or spontaneous games and recreational activities also form the foundation of sports participation, introducing children to the values of cooperation, fair play, and discipline. Oliver et al. (2010) highlighted the importance of early education in promoting lifelong physical activity habits in children and stressed the necessity of providing equitable access to appropriate infrastructure and facilities.

Within this framework, recreationally oriented exercise programs can offer a practical and effective strategy for addressing both digital addiction and sleep-related problems among children. Regular participation in enjoyable physical activities may help reduce the time allocated to screen-based behaviors, increase overall energy expenditure, and contribute to the regulation of biological rhythms, including sleep–wake cycles. Moreover, group-based recreational exercises can facilitate peer interaction, strengthen social bonds, and enhance children’s sense of belonging and self-efficacy, thereby providing psychosocial benefits that may indirectly reduce reliance on digital environments for social gratification. Nevertheless, despite the theoretical plausibility and practical appeal of such interventions, empirical research on the long-term effects of structured recreational exercise programs on both digital addiction and sleep quality in children remains limited. A key theoretical lens through which these dynamics can be understood is the Displacement Hypothesis, which posits that time and cognitive resources are finite, and that engagement in one activity necessarily reduces the time available for competing behaviors (Mutz et al., 1993). Applied to the present context, this framework suggests that structured physical activity does not merely add an additional behavior to a child’s daily routine but actively displaces sedentary screen-based engagement by occupying the temporal, energetic, and motivational space that digital media would otherwise fill. When children are regularly engaged in enjoyable, socially rewarding physical activities, the opportunity cost of digital media use increases, and the behavioral and psychological pull of screens is correspondingly diminished. This theoretical position aligns with observations that children who participate consistently in organized physical activities tend to report lower levels of problematic digital use (Hazar et al., 2017; Can and Tekkurşun Demir, 2020). The present study adopts the Displacement Hypothesis as its primary theoretical framework, testing whether a structured recreational exercise program can serve as an effective behavioral substitute for screen-based leisure in early adolescents.

The primary aim of the present study is to experimentally examine the effects of an eight-week recreationally oriented regular exercise program on digital addiction levels and sleep quality in children aged 10–14 years. By simultaneously addressing these two interrelated domains, the study seeks to provide a more holistic understanding of how movement-based recreational activities can contribute to children’s biopsychosocial well-being. The intervention is designed to offer age-appropriate, enjoyable, and socially engaging physical activities that can be feasibly integrated into school or community settings. A further distinctive feature of the study is the inclusion of a follow-up assessment to evaluate the persistence of the intervention effects over time, which is often lacking in the existing literature. Based on the theoretical framework outlined above and the existing empirical literature, the following hypotheses were formulated: (H1) Children in the recreational exercise group will demonstrate significantly greater reductions in digital addiction scores from pre-test to post-test compared to the control group; (H2) Children in the recreational exercise group will demonstrate significantly greater improvements in sleep quality scores from pre-test to post-test compared to the control group; (H3) The intervention-related improvements in both digital addiction and sleep quality will be maintained at the six-week follow-up assessment, indicating durable behavioral change. In this regard, the present research aims to fill a critical gap by providing experimental evidence on the potential of recreational exercise programs to reduce digital addiction and improve sleep quality in early adolescents.

Based on the findings obtained, this study is expected to offer concrete, evidence-based recommendations for the development of school-based and community-based intervention programs targeting digital addiction and sleep problems in children. The results may guide educators, policymakers, mental health professionals, and practitioners working in sports and recreation in designing comprehensive strategies that integrate physical activity, psychoeducation, and parental involvement. By emphasizing the protective role of recreational exercise and play, the study seeks to contribute to the growing body of research focused on promoting healthy technology use, enhancing sleep hygiene, and supporting overall well-being in children during a critical developmental period.

## Materials and methods

### Study group

The study group was determined using a purposive sampling method followed by a rigorous screening process. Initially, a volunteer pool of 126 middle school students (56 girls and 70 boys) aged 10–14 years was formed during the 2025–2026 academic year in Konya, Turkiye. To ensure the homogeneity of the groups, students with similar baseline body mass index (BMI), calculated from height and weight measurements, were prioritized. Participants were included if they met the following eligibility criteria: (a) aged between 10 and 14 years, (b) enrolled in middle school during the 2025–2026 academic year, (c) obtaining a score at or above the scale midpoint on the Digital Addiction Scale for Adolescents at baseline screening, and (d) written informed consent provided by a parent or legal guardian. Participants were excluded if they: (a) had a previously diagnosed sleep disorder (e.g., insomnia, sleep apnea, restless leg syndrome), clinical depression, attention-deficit/hyperactivity disorder (ADHD), or any other psychiatric condition that could independently influence sleep or addictive behavior; (b) were currently receiving pharmacological or psychological treatment for any behavioral or mental health condition; (c) were regularly participating in an organized, coach-led sport or physical activity program outside of school (defined as two or more structured sessions per week) at the time of recruitment, as this could attenuate the expected response to the intervention; or (d) had any physical condition or injury that would prevent full participation in moderate-to-vigorous physical activity. These criteria were verified through a structured parent-report screening questionnaire administered prior to randomization.

A power analysis was conducted using G*Power (version 3.1.9.7) to determine the required sample size. For a 2×3 mixed-design ANOVA with an effect size of *f* = 0.25 (medium effect), an alpha level of 0.05, and a power of 0.80, the minimum required sample size was calculated as 34. To account for potential dropouts, 40 participants were included. To ensure the integrity of the dose–response relationship, a minimum attendance threshold was established *a priori*: participants were required to attend at least 14 of the 18 scheduled sessions (≥78%) to be included in the final analyses. Attendance was recorded at every session by the supervising physical education teacher. Of the 40 participants enrolled at baseline, all 40 completed the 8-week intervention and the 6-week follow-up assessment, yielding a dropout rate of 0%. All participants met the minimum attendance criterion; mean session attendance in the REG was 17.2 out of 18 sessions (SD = 0.6), indicating a high level of engagement throughout the program.

Participants were allocated into two parallel groups: the Recreational Activity Group (RAG, *n* = 20) and the Control Group (CG, *n* = 20). To eliminate selection bias, a stratified block randomization procedure was employed. Students were first stratified by gender (20 boys, 20 girls) and then by age groups (10–12 and 13–14). Within each stratum, a computer-generated random number sequence (using Research Randomizer software) was used to assign participants to either the RAG or CG.

To ensure the integrity of the “blind” assignment, the allocation was performed by an independent researcher not involved in the data collection or the implementation of the intervention. The ratios were kept at 1:1, ensuring an equal distribution of 10 boys and 10 girls in each group.

### The recreational activity program (intervention)

The intervention for the RAG was designed as a multi-component program focusing on physical movement, social interaction, and cognitive engagement through play (see Table 1). The program was implemented over 8 weeks, consisting of a two-week familiarization phase followed by a six-week active intervention period.

**Table 1:** Weekly recreational activity program content.

Week	Activities
1 (Activities 1–3)	Skittles throwing, street basketball, darts
2 (Activities 4–6)	Balloon popping on chairs, four-a-side football, bocce
3 (Activities 7–9)	Hoop throwing, volleyball, kite flying
4 (Activities 10–12)	Obstacle courses and races, handball, hopscotch
5 (Activities 13–15)	Target games, table tennis, musical chairs and tag games
6 (Activities 16–18)	Task-based wheel game, dodgeball, outdoor marble games

Each of the 18 sessions (3 days/week) was structured into three distinct phases to ensure physiological safety and psychological readiness:

1. Warm-up Phase (10–15 min): Low-intensity aerobic activities and dynamic stretching to prepare the musculoskeletal system.
2. Main Activity Phase (35–40 min): High-engagement games and sports (as detailed in Table 1). This phase aimed for moderate-to-vigorous physical activity (MVPA), targeting a heart rate of approximately 60–75% of the age-predicted maximum.
3. Cool-down Phase (5–10 min): Breathing exercises, static stretching, and a brief verbal reflection on the day’s activities to facilitate recovery and social bonding.

The program was executed in the school’s indoor sports hall and outdoor playground under the supervision of a certified physical education teacher and two subject-area instructors. Prior to the commencement of the program, the certified physical education teacher and both subject-area instructors participated in a two-session orientation (totaling approximately 4 h) covering the core principles of the Teaching Games for Understanding (TGfU) model, including the prioritization of tactical awareness, intrinsic motivation, and inclusive participation over competitive performance outcomes. To ensure fidelity of implementation across all 18 sessions, a standardized session checklist was developed based on the program protocol. For each session, the supervising physical education teacher completed this checklist, documenting whether the warm-up, main activity, and cool-down phases were delivered as planned, whether the target heart rate zone (60–75% of age-predicted maximum) was approximately achieved, and whether any deviations from the planned activities occurred. Additionally, five sessions (approximately 28% of all sessions) were independently observed by a researcher not involved in the delivery of the intervention, who used the same checklist to assess adherence. Inter-rater agreement between the session supervisor and the independent observer was calculated and found to be satisfactory (Cohen’s *κ* > 0.80), confirming that the program was delivered with a high degree of consistency throughout the intervention period.

Activities were adjusted according to the “Teaching Games for Understanding” (TGfU) model, emphasizing tactical awareness and enjoyment over pure competition. Meanwhile, the Control Group (CG) continued their standard school curriculum without participating in any additional organized physical or recreational activities. To verify that the control group did not independently increase their physical activity levels during the study period—which could have confounded between-group comparisons—CG participants were asked to complete a brief weekly physical activity log throughout the 8-week intervention. The log recorded the type, frequency, and approximate duration of any leisure-time physical activity undertaken outside of regular school physical education classes. Weekly logs were collected by a physical education teacher at the end of each school week. Review of the completed logs indicated no systematic increase in leisure-time physical activity among CG participants during the intervention period, supporting the assumption that observed between-group differences were attributable to the recreational exercise program rather than differential changes in habitual activity.

### Data collection instruments

A personal information form developed by the researchers was used to collect socio-demographic data. Digital addiction levels were assessed using the Digital Addiction Scale for Adolescents, while sleep quality was evaluated using the Sleep Quality and Sleep Variables Scale. All measurement tools were administered to both the Recreational Activity Group (RAG) and the Control Group (CG) at three time points: before the intervention (pre-test), immediately after the intervention (8th week; post-test), and 6 weeks after the completion of the intervention (follow-up).

Several contextual and environmental factors with potential to confound sleep-related outcomes were considered in the design of the study. The intervention was conducted during a single, continuous 8-week period within the same academic semester, thereby minimizing exposure to major academic stressors such as high-stakes examination periods, which are known to disrupt adolescent sleep patterns. Specifically, the intervention was scheduled to avoid the semester-end examination weeks at the participating school. Furthermore, as all measurement points, pre-test, post-test, and follow-up, were completed within the same academic year and season, systematic variation in ambient daylight hours, which can influence circadian rhythms and sleep–wake cycles in adolescents, was minimized. Both the experimental and control groups were drawn from the same school and geographic region, ensuring equivalent exposure to any residual seasonal or environmental influences that could not be experimentally controlled.

### Digital addiction scale for adolescents

The Digital Addiction Scale for Adolescents was originally developed by Seema et al. (2022) and later adapted into Turkish by Çelik et al. (2023). The scale has a unidimensional structure and consists of 10 items rated on a 5-point Likert scale ranging from 1 (Never) to 5 (Always). Total scores range from 10 to 50, with higher scores indicating higher levels of digital addiction. In the Turkish adaptation study, the internal consistency coefficient of the scale was reported as Cronbach’s *α* = 0.90.

### Sleep quality scale and sleep variables questionnaire

Sleep quality and sleep-related variables were assessed using the scale developed by Meijer and Van den Wittenboer (2004) and adapted into Turkish by Önder et al. (2016). The scale consists of 15 items in total. The first seven items assess subjective sleep quality, while the remaining eight items evaluate sleep-related variables such as total sleep duration, sleep efficiency, and parental control. In the Turkish adaptation study, the internal consistency coefficient of the sleep quality subscale was reported as Cronbach’s *α* = 0.72.

### Statistical analysis

All statistical analyses were conducted using IBM SPSS Statistics (version 27). Prior to the main analyses, the data were screened for missing values and outliers. Descriptive statistics (mean, standard deviation, minimum, and maximum values) were calculated for all variables.

The assumptions of normality were evaluated by examining skewness and kurtosis values. Values within the acceptable range of ±2 were considered indicative of an approximately normal distribution (George and Mallery, 2010). Homogeneity of variances was assessed using Levene’s test. No substantial violations of the assumptions were detected; therefore, parametric statistical procedures were applied.

To examine the effects of group (experimental vs. control) and time (pre-test, post-test, follow-up) on the dependent variable, a 2 × 3 mixed-design analysis of variance (ANOVA) was conducted, with group as the between-subjects factor and time as the within-subjects factor. The group × time interaction effect was primarily examined to determine whether changes across measurement occasions differed between groups. This analytical approach was directly aligned with the *a priori* hypotheses of the study. The Time × Group interaction effect was the primary test of H1 and H2, examining whether reductions in digital addiction and improvements in sleep quality were significantly greater in the REG compared to the CG. The follow-up pairwise comparisons between post-test and follow-up scores served as the primary test of H3, evaluating whether intervention gains were maintained over the six-week post-intervention period.

When significant main or interaction effects were observed, *post hoc* pairwise comparisons with Bonferroni adjustment were performed to identify the source of the differences. Effect sizes were reported using partial eta squared (*η*^2^*p*) and interpreted according to the criteria suggested by Cohen (1988) (small = 0.01, medium = 0.06, large = 0.14).

In addition, estimated marginal means were calculated, and the findings were supported by tables and graphical representations illustrating changes in mean scores across time and groups. The level of statistical significance was set at *p* < 0.05.

## Results

The descriptive statistics (Table 2) reveal a distinct divergence in the trajectories of the two groups. At the baseline (Pre-test), both the Experimental (RAG) and Control (CG) groups exhibited nearly identical mean scores for digital addiction (42.30 ± 0.55 vs. 42.80 ± 0.55) and sleep quality (13.90 ± 0.47 vs. 13.85 ± 0.47), confirming the effectiveness of the initial randomization.

**Table 2:** Descriptive statistics for digital addiction and sleep quality.

Variable	Group	Pre-test (*M* ± SE)	Post-test (*M* ± SE)	Follow-up (*M* ± SE)
Digital addiction	Experimental	42.30 ± 0.55	38.65 ± 0.57	39.15 ± 0.49
	Control	42.80 ± 0.55	42.40 ± 0.57	42.05 ± 0.49
Sleep quality	Experimental	13.90 ± 0.47	10.95 ± 0.27	11.40 ± 0.25
	Control	13.85 ± 0.47	13.45 ± 0.27	13.50 ± 0.25

M, mean; SE, standard error. Lower sleep quality scores represent better sleep quality.

However, following the six-week intervention, the Experimental group showed a precipitous decline in digital addiction levels, whereas the Control group’s scores remained virtually stagnant. A similar trend was observed for sleep quality; considering that lower scores represent improved sleep, the RAG demonstrated a significant enhancement in sleep health, while the CG showed no clinically relevant change.

The 2 (Group) x 3 (Time) Mixed-Design ANOVA results (Table 3) indicate that the intervention had a profound impact on the participants. A significant Time x Group interaction was found for digital addiction, *F*(2, 76) = 18.98, *p* < 0.001, *η*^2^_p_ = 0.333. This interaction suggests that the change in digital addiction over time was fundamentally different between the two groups. The partial eta squared value (*η*^2^_p_ = 0.333) indicates a large effect size, suggesting that approximately 33.3% of the variance in digital addiction scores can be explained by the intervention’s interaction with time.

**Table 3:** Repeated measures ANOVA results.

Variable	Effect	*F*	df	*p*	Partial *η*^2^
Digital addiction	Time	35.23	2, 76	<0.001	0.481
	Time × Group	18.98	2, 76	<0.001	0.333
Sleep quality	Time	21.20	2, 76	<0.001	0.358
	Time × Group	12.21	2, 76	0.001	0.243

Significant interactions indicate differential change patterns between groups over time.

Similarly, for sleep quality, a significant interaction effect was observed, *F*(2, 76) = 12.21, *p* = 0.001, *η*^2^_p_ = 0.243. This also represents a large effect size, highlighting that the recreational activity program was a primary driver in the divergence of sleep quality trajectories between the groups.

**Figure 1:**
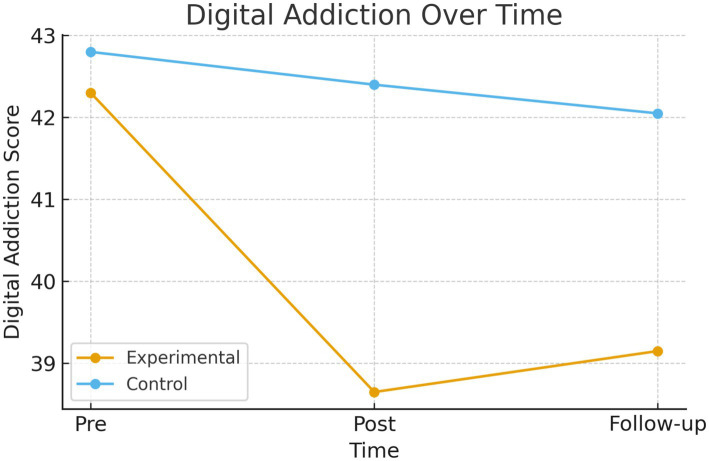
Digital addiction over time. Experimental group showed a significant reduction from pre-test to post-test, maintained at follow-up.

**Figure 2:**
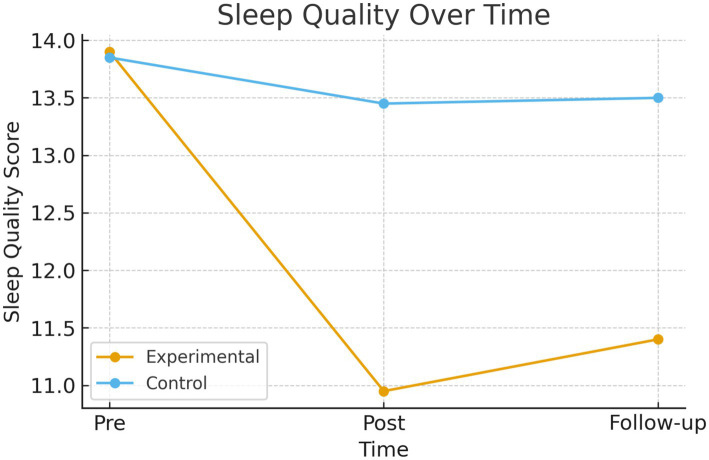
Sleep quality over time. Lower scores represent better sleep quality. Experimental group demonstrated marked improvement from pre-test to post-test.

The visual representation of these findings in Figures 1, 2 further elucidates the impact of the program.

Digital Addiction (Figure 1): The RAG demonstrated a sharp negative slope between the pre-test and post-test, indicating a rapid reduction in addiction levels. In contrast, the CG’s slope remained nearly horizontal (zero slope), representing stability in their addiction levels. The “gap” between the two groups widened significantly at the 8th week (Post-test) and remained wide at the 14th week (Follow-up), suggesting that the intervention’s effects did not dissipate immediately after the program ended.

Sleep Quality (Figure 2): The RAG showed a significant downward trajectory (improvement), with the most substantial gains occurring during the active intervention period. The Control group, however, maintained a high-score plateau across all measurements. The increasing distance between the two lines over time illustrates that the intervention successfully “decoupled” the experimental participants from the poor sleep quality patterns observed in their peers.

As shown in the pairwise comparisons (Table 4), the improvements achieved by the Experimental group were not transient. For digital addiction, the mean reduction from pre-test to post-test was 2.025 units (*p* < 0.001), and this gain was sustained at the follow-up measurement, as the difference between post-test and follow-up was statistically negligible (*p* = 1.00).

**Table 4:** Pairwise comparisons.

Variable	Comparison	Mean difference	*p*
Digital addiction	Pre → Post	2.025	<0.001
	Pre → Follow-up	1.950	<0.001
	Post → Follow-up	0.075	1.000
Sleep quality	Pre → Post	1.675	<0.001
	Pre → Follow-up	1.425	<0.001
	Post → Follow-up	0.250	0.449

Bonferroni correction applied.

For sleep quality, the intervention resulted in a significant improvement of 1.675 units from pre-test to post-test (*p* < 0.001). Although a slight fluctuation was observed during the follow-up period (Table 2), the pairwise comparison in Table 4 confirms that the difference between post-test and follow-up was not significant (Mean Difference = 0.250, *p* = 0.449). This indicates that the participants continued to experience significantly better sleep quality than their baseline levels even 6 weeks after the recreational activities had ceased.

## Discussion

This study is an experimental investigation examining the effects of a structured recreational activity program on digital addiction levels and sleep quality among middle school students aged 10–14 years. Overall, the findings largely support the main assumptions of the intervention. The results of the repeated-measures ANOVA revealed significant changes over time in both digital addiction and sleep quality, with these changes differing significantly between groups (Time × Group interaction, *p* < 0.001). This finding indicates that the observed improvements were primarily attributable to participation in the recreational activity program. Considered in relation to the study’s *a priori* hypotheses, all three predictions were supported by the data. H1 and H2 were confirmed by the significant Time × Group interaction effects for both digital addiction (*η*^2^_p_ = 0.333) and sleep quality (*η*^2^_p_ = 0.243), indicating that the REG demonstrated substantially greater improvements than the CG across the intervention period. H3 was likewise supported: pairwise comparisons revealed no statistically significant difference between post-test and follow-up scores for either outcome, confirming that the gains achieved during the active intervention phase were maintained 6 weeks after the program concluded.

In the recreational activity group, digital addiction scores significantly decreased from pre-test to post-test, and this reduction was maintained at the follow-up measurement. Pairwise comparisons demonstrated significant differences between pre-test and post-test, as well as between pre-test and follow-up, whereas no significant difference was observed between post-test and follow-up. These results suggest that the beneficial effects of the intervention were sustained over time. The findings are consistent with previous studies reporting that recreational and physical activities contribute to reductions in addictive behaviors (Hazar et al., 2017; Keskin et al., 2021; Alshehri and Mohamed, 2019). Similarly, research indicating that participation in sports activities is associated with lower levels of digital addiction further supports the present results (Ekinci et al., 2017; Can and Tekkurşun Demir, 2020; Güvendi et al., 2019).

The protective functions of recreational activities—such as promoting active recovery, reducing stress, and enhancing social interaction—may help explain these outcomes (Caldwell, 2005). The game-based and socially interactive activities implemented in the present program may have transferred the psychosocial needs that children often attempt to fulfill in digital environments into real-life contexts, thereby reducing reliance on digital media (Çömlekçi and Başol, 2019; Ece et al., 2023). While the overall effectiveness of the program is well-supported by the present findings, the multi-component nature of the intervention warrants a more nuanced consideration of which specific elements may have driven the observed outcomes. The recreational activity program simultaneously incorporated aerobic physical exertion, structured social interaction, cognitive engagement through game-based tasks, and a reduction in available screen time—each of which represents a plausible mechanism of change.

With respect to digital addiction, the social interaction dimension of the program is a particularly compelling candidate. The group-based format of the sessions provided children with a direct, real-life context for fulfilling the social and emotional needs such as belongingness, peer recognition, and cooperative engagement that are frequently sought through digital environments (Çömlekçi and Başol, 2019). If social gratification was indeed the primary mechanism, one would expect low-intensity but socially rich activities (e.g., cooperative games, team-based tasks) to produce comparable reductions in digital craving as high-intensity aerobic activities. Conversely, if aerobic demand was the primary driver, the physiological effects of exercise on dopaminergic reward pathways which overlap with the neurobiological substrates of behavioral addiction may have reduced the salience of digital stimuli by providing an alternative source of reward (Dishman et al., 2006).

With respect to sleep quality, the two mechanisms are similarly difficult to disentangle. The aerobic components of the program likely contributed through the thermoregulatory and adenosine-mediated pathways described previously, whereas the social and emotionally fulfilling nature of the activities may have reduced pre-sleep cognitive arousal and rumination, a well-documented barrier to sleep onset in adolescents (Harvey, 2002; Liu et al., 2025). It is also plausible that the displacement of screen time itself, rather than any direct physiological or psychosocial effect of exercise, was the proximal mechanism for sleep improvement: by occupying afternoon hours with physical activity, the program may have structurally reduced late-evening device use and its associated blue-light exposure.

The present study design does not permit a definitive attribution of effects to any single component, and this remains an important limitation. Nevertheless, it is reasonable to propose that the observed outcomes were most likely the product of a synergistic interaction among aerobic demand, social engagement, cognitive stimulation, and behavioral displacement, rather than the result of any isolated ingredient. Future dismantling studies that systematically vary these components will be essential for understanding the relative contribution of each mechanism and for designing more targeted and resource-efficient interventions. This interpretation aligns with studies demonstrating that learning through play contributes positively to children’s cognitive and social development (Parker et al., 2022; Güzel Gürbüz et al., 2024).

The age range of 10–14 years represents a critical developmental period characterized by increased vulnerability to digital addiction. During this stage, children frequently experience academic challenges, communication difficulties, and problems related to responsibility management (Koçak and Köse, 2014; Kuss and Griffiths, 2012). Studies showing heightened tendencies toward addictive behaviors during adolescence underscore the importance of early preventive interventions (Gentile, 2009; Irmak and Erdoğan, 2016; Bayrak and Demirel, 2023). In this regard, the present findings suggest that recreational interventions may serve as an effective preventive strategy during this sensitive developmental window.

Findings related to sleep quality also support the positive impact of the recreational activity program. In the recreational activity group, sleep quality scores improved significantly over time, and these improvements were maintained at follow-up. These results are consistent with studies highlighting the close relationship between digital addiction and sleep disturbances (De Pasquale et al., 2020; Özgür, 2019). Participation in outdoor and physical activities may have contributed to improved sleep by reducing stress levels and enhancing emotional well-being (Schneider et al., 2024; Vasilaki et al., 2025).

## Conclusion

In conclusion, the present study provides robust empirical evidence that regular participation in structured recreational physical activities represents a highly effective, non-pharmacological, and practical approach to mitigating digital addiction and enhancing sleep quality among middle school students. The results underscore a significant divergence between the intervention and control groups; while the recreational activity group (RAG) demonstrated substantial improvements from pre-test to post-test, the control group remained stagnant, highlighting that such behavioral shifts are unlikely to occur spontaneously without organized intervention.

Crucially, the maintenance of these gains during the six-week follow-up period suggests that the intervention triggered a durable behavioral modification rather than a transient effect. This durability is particularly vital during early adolescence, a critical developmental window characterized by neurobiological maturation and the formation of lifelong lifestyle habits. From a theoretical perspective, these findings support the “Displacement Hypothesis,” suggesting that providing high-engagement physical alternatives effectively displaces sedentary screen time, thereby recalibrating the participants’ daily routines and physiological rhythms. This interpretation is consistent with the theoretical position established in the introduction of the present study. As outlined therein, the Displacement Hypothesis does not simply predict a reduction in screen time as a byproduct of busyness; rather, it proposes a more fundamental reallocation of motivational and attentional resources. When children are regularly engaged in activities that fulfill their social, emotional, and physiological needs, as the present recreational program was designed to do, the relative reward value of digital stimuli diminishes. The maintenance of improvements at the six-week follow-up is particularly noteworthy in this regard: it suggests that the behavioral reallocation triggered by the intervention was not merely a temporary displacement during the program weeks, but reflected a more durable restructuring of children’s leisure-time preferences and daily routines. This finding lends empirical support to the Displacement Hypothesis as a viable theoretical framework for designing and evaluating physical activity–based interventions targeting digital dependency in early adolescence.

Accordingly, the holistic impact of these programs on physical health, psychological resilience, and social connectedness positions them as essential tools for school-based preventive health strategies. It is imperative for school administrations, policymakers, and families to move beyond traditional academic frameworks and integrate extracurricular recreational activities as a core component of the curriculum. Such an integration would not only address the academic (Sun et al., 2023) and social challenges (Koçak and Köse, 2014) associated with digital over-dependence but also foster a sustainable environment for the overall well-being and quality of life of the digital-native generation.

### Limitations and future directions

Despite the significant insights gained, the present study has several limitations that provide a roadmap for future scholarly inquiry. First, the relatively small sample size (*N* = 40) and the specific geographical focus on a single region limit the broader generalizability of the findings. While the randomized controlled trial (RCT) design and the inclusion of a follow-up assessment ensure high internal validity, the external validity of these results remains to be tested across more diverse socio-economic, cultural, and regional contexts. Future research should employ larger, multi-center cohorts to confirm these effects across different demographic strata.

Second, the assessment of digital addiction and sleep quality relied exclusively on self-report questionnaires. Although the instruments utilized—the Digital Addiction Scale for Adolescents and the Sleep Quality Scale—have demonstrated high psychometric reliability, they remain susceptible to social desirability bias and retrospective recall errors common in adolescent populations. To achieve a more granular and objective understanding of these phenomena, future studies should incorporate technological assessments, such as ecological momentary assessment (EMA) through screen-time tracking applications, and actigraphy-based devices for precise sleep architecture monitoring.

Third, although the six-week follow-up period confirmed the short-term durability of the intervention, it does not account for long-term behavioral trajectories. Digital addiction patterns often fluctuate with seasonal changes, academic exam periods, or shifts in social media trends. Subsequent longitudinal research should extend the follow-up duration to 6 months or a year to determine if the “recreational habit” remains resilient against the increasing pressures of the digital landscape.

Furthermore, the intervention utilized a general, multi-component recreational program. While effective, this design does not allow for the isolation of specific “active ingredients” of the program. Future investigations should conduct comparative analysis between different modalities—such as high-intensity team sports versus low-intensity individual outdoor activities—to identify which components (e.g., social interaction, aerobic demand, or cognitive challenge) contribute most significantly to the reduction of digital cravings.

Finally, future research should delve into the underlying psychological and neurobiological mechanisms. Variables such as executive function, emotional regulation, and dopamine-related reward processing may play mediating roles in how recreational activities counteract the addictive nature of digital stimuli. A mixed-methods approach, combining quantitative data with qualitative interviews, could provide a deeper narrative on the participants’ subjective experiences of “switching off” and “plugging into” physical play.

## Data Availability

The raw data supporting the conclusions of this article will be made available by the authors, without undue reservation.
